# Very rare myoepithelial carcinoma occurred in the humerus: a case report

**DOI:** 10.1097/RC9.0000000000000282

**Published:** 2026-02-16

**Authors:** Taisuke Furuta, Tomohiko Sakuda, Koki Yoshioka, Koji Arihiro, Nobuo Adachi

**Affiliations:** a Department of Orthopaedic Surgery, Hiroshima University, Graduate School of Biomedical and Health Sciences, Hiroshima, Japan; b Department of Pathology, Hiroshima University Hospital, Hiroshima, Japan

**Keywords:** bone tumor, case report, humerus, limb salvage, myoepithelial carcinoma

## Abstract

**Introduction:**

Myoepithelial carcinoma (MEC) most frequently arises in the salivary glands; intraosseous disease is exceptionally rare. Early recognition is challenging, and evidence‑based management is lacking.

**Case presentation:**

A 16‑year‑old Japanese male presented with left shoulder pain. A proximal humeral lesion was monitored using radiographs over a 2-year period, after which an urgent biopsy identified MEC. He received two cycles of paclitaxel (200 mg/m^2^) plus carboplatin (AUC 5) as neoadjuvant chemotherapy, followed by wide resection and vascularized fibular reconstruction. Margins were negative.

**Discussion:**

Despite preoperative and postoperative adjuvant chemotherapy, the patient developed lung metastasis 1 year after surgery and died 6 months later. Delayed biopsy may have contributed to disease progression. Current literature recommends early biopsy, extensive resection, and consideration of molecular targeted therapy.

**Conclusion:**

Bone MEC is aggressive; prompt histological diagnosis and multidisciplinary treatment are crucial to improve prognosis.

## Introduction

Myoepithelial carcinoma (MEC) is a rare but distinctive malignant tumor accounting for less than 2% of all salivary gland cancers[1]. Most cases arise as malignant transformations or new occurrences of pleomorphic adenomas within major or minor salivary glands; however, recent studies have reported MEC arising in non-salivary gland sites such as the bladder, paranasal sinuses, lungs, and breasts, emphasizing its morphological plasticity and potential for ectopic development[2]. Within this already rare spectrum, the osteogenic (intraosseous) variant is extremely rare and highly malignant, with only isolated case reports or small series reported to date[1]. Due to its low incidence and clinical and pathological heterogeneity, early recognition is difficult and often leads to long-term follow-up or misclassification as a benign bone tumor. Delayed diagnosis may have contributed to disease progression.

Intraosseous MEC is extremely rare, and standard treatment algorithms have not been established. Complete *en bloc* surgical resection with clear margins is the cornerstone of treatment and the most consistent predictor of local control[3]. However, due to the complex anatomy of long bones and the risk of neurovascular compromise, achieving wide margins is often challenging, necessitating consideration of multimodal therapy .In recent series, adjuvant external beam radiation therapy, conventional cytotoxic chemotherapy, or, in selected cases, molecularly targeted agents targeting EGFR, HER2, or other actionable pathways have been recommended^[^4–6^]^. Recent reports have also described cases where intensity-modulated proton beam therapy and immune checkpoint inhibitors were effective in unresectable or metastatic cases^[^7,8^]^. However, due to a tendency toward late local recurrence and distant metastasis, the 5-year survival rate is estimated to be 40%–60%[3], and the long-term prognosis remains guarded.

**HIGHLIGHTS**

- An extremely rare case of intraosseous myoepithelial carcinoma of the humerus.
- Initially misdiagnosed as benign and followed radiographically for 2 years.
- Diagnosis confirmed by biopsy showing biphasic epithelial–myoepithelial features.
- Wide resection with vascularized fibular graft reconstruction achieved limb salvage.
- Case emphasizes early biopsy and multidisciplinary management for rare bone tumors.

This report presents a case of humeral origin MEC that was diagnosed after being observed as a benign bone lesion for 2 years. The authors clarify the radiological and histopathological pitfalls encountered during the diagnostic process and outline a multidisciplinary treatment strategy. Additionally, the authors integrate a focused literature review to summarize the current evidence. This work has been reported in line with the SCARE 2025 criteria[9].

## Presentation of case

**Figure 1.**
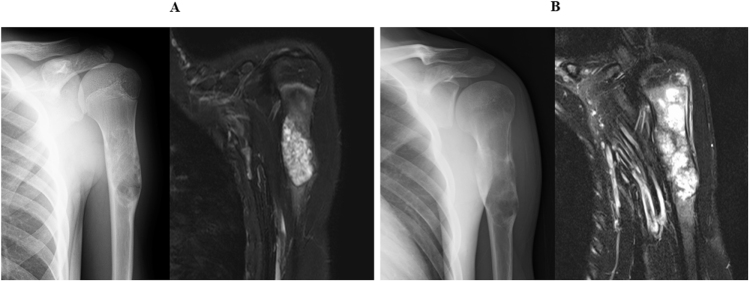
Radiographic and MRI findings at the initial visit and after 2 years of follow-up. (A) Plain radiograph and MRI at the initial presentation. (B) Follow-up imaging 2 years later, showing interval changes.

A 16-year-old Japanese male presented with a complaint of left shoulder pain. His medical, family, and medication histories were unremarkable. Plain radiographs obtained at the initial visit revealed a bone lesion in the proximal humerus. Based on the radiographic and magnetic resonance imaging (MRI) findings, the initial treatment physician suspected a benign to intermediate-grade bone tumor. Accordingly, the lesion was followed with serial radiographs every few months for 2 years without intervention (Fig. 1A).

**Figure 2.**
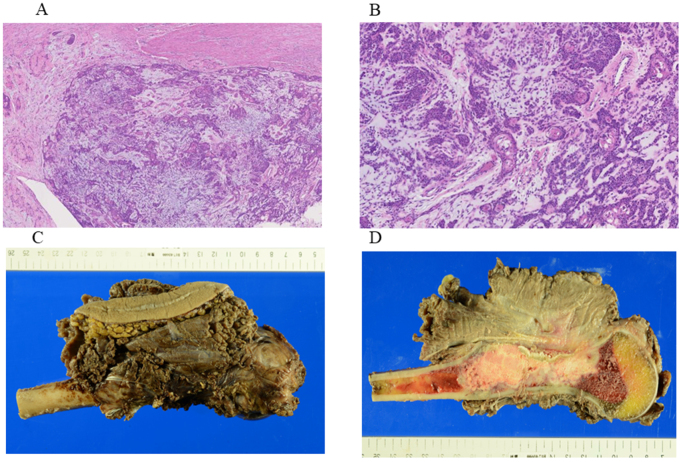
Histopathological examination and gross morphology of the resected tumor. (A, B) Hematoxylin and eosin staining. Original magnifications: (A) × 100, (B) × 400. (C, D) Gross appearance of the excised humeral tumor. (C) Resected specimen. (D) Longitudinal section through the bone and tumor mass.

Following a change in attending physicians, the authors’ team assumed care of the patient. At that time, radiographs demonstrated progressive enlargement of the lesion. Based on these findings, differential diagnoses included giant cell tumor of bone, aneurysmal bone cyst, and malignant bone tumor (Fig. 1B). An urgent biopsy was performed. Histopathological analysis of the humeral tumor revealed a biphasic architecture composed of inner tubular epithelial cells and outer myoepithelial cells with clear to faintly eosinophilic cytoplasm (Fig. 2A, B). Immunohistochemically, the tumor was positive for S-100, epithelial membrane antigen, smooth muscle actin (SMA), cytokeratin AE1/AE3, and p63. The Ki-67 labeling index was high at 69%, supporting a diagnosis of MEC.

**Figure 3.**
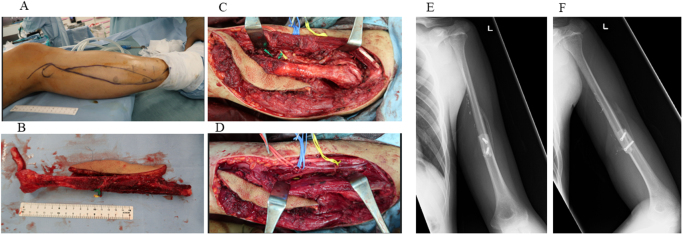
Upper limb reconstruction using a vascularized fibular graft. (A) Right lower leg prior to graft harvesting. (B) Harvested fibular graft with vascular pedicle (donor site). (C) Fixation of the fibula to the recipient site. (D) Microvascular anastomosis between the fibular vessels and the brachial artery and vein. (E) Postoperative anteroposterior radiograph. (F) Postoperative lateral radiograph.

**Figure 4.**
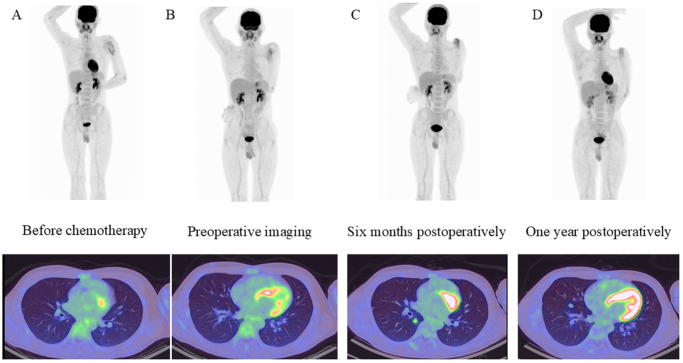
Sequential PET-CT images showing tumor metabolic activity. (A) Before chemotherapy. (B) Preoperative imaging. (C) Six months postoperatively. (D) One year postoperatively. PET-CT, positron emission tomography-computed tomography.

Whole-body CT scanning showed no evidence of a primary lesion in the head and neck region, which is a common origin for this tumor, nor were any distant metastases identified. After multidisciplinary discussion with medical oncology and head and neck surgery teams, neoadjuvant chemotherapy was initiated due to the prolonged natural course of the disease:

Two cycles of paclitaxel (200 mg/m^2^) and carboplatin (AUC 5), administered on day 1 of each 21-day cycle, were given as neoadjuvant therapy. Given the extensive involvement of the lesion requiring a wide resection of the humerus, limb preservation was pursued through planned reconstruction with a vascularized fibular graft. A deltopectoral approach was used with a skin incision extended distally. The tumor had eroded the anterior cortex and extended into the surrounding soft tissue. Infiltrated portions of the brachialis, biceps, and triceps muscles were resected en bloc with the tumor to preserve surgical margins. The proximal humerus was osteotomized 18 cm distal to the humeral head, with a 5-cm margin from the tumor. A sufficient resection margin was secured (Fig. 2C, D). A 25-cm segment of the right fibula was harvested with its vascular pedicle (peroneal artery and veins). Microvascular anastomoses were performed to the circumflex humeral artery and two accompanying veins to restore circulation. The distal humerus was partially reamed using a burr to accommodate the fibular graft, which was inserted intramedullary. Two 3.5-mm cortical screws (Meira, Japan) were inserted laterally to secure the overlapping portion of the graft (Fig. 3). Following surgery, the patient underwent adjuvant chemotherapy: Three cycles of paclitaxel (200 mg/m^2^) and carboplatin (AUC 5), administered on day 1 of each 21-day cycle, were given postoperatively. Histological examination of the resected specimen confirmed negative surgical margins, and therefore no postoperative radiotherapy was administered. The patient remained free of local recurrence or distant metastasis based on routine follow-up examinations until the 1-year postoperative positron emission tomography-computed tomography, which subsequently revealed pulmonary metastases (Fig. 4). After consultation with medical oncology and otolaryngology, the patient received immune checkpoint inhibitor therapy: Nivolumab was administered at a fixed dose of 240 mg every 2 weeks (q2w) for six cycles. Despite treatment, the metastatic disease progressed, and the patient subsequently passed away 16 months postoperatively.

## Discussion

MEC is a rare malignant tumor that presents significant diagnostic and therapeutic challenges, particularly when it arises in bone. Its rarity, histological heterogeneity, and non-specific radiographic features often lead to misdiagnosis and delayed treatment. In the present case, the biopsy was delayed because the initial radiographic and MRI findings suggested a benign to intermediate-grade bone tumor, leading the initial treating physician to pursue a 2-year period of radiological follow-up without immediate intervention. This highlights a critical pitfall; tissue diagnosis should be prioritized early when a bone lesion demonstrates progressive growth or cortical destruction. Mahdi *et al* emphasized that MEC may closely mimic other sarcomas or carcinomas histologically, further complicating the diagnostic process[10]. Additionally, as Zhang *et al* noted, the limited clinical familiarity with MEC among general practitioners and pathologists contributes to diagnostic delays[11].

Histologically, intraosseous MEC demonstrates dual epithelial and myoepithelial differentiation, typically forming tubular or spindle-cell structures within a sclerotic stroma. Malignant features such as nuclear pleomorphism, brisk mitotic activity, and focal necrosis have been described in prior reports^[^12,13^]^. Immunohistochemically, most tumors show broad cytokeratin positivity together with S-100 protein and SMA, with variable expression of GFAP or p63, a profile that helps distinguish MEC from other primary bone tumors^[^2,12,13^]^.

Recent molecular studies in soft-tissue and salivary-type MEC have identified recurrent EWSR1-related fusions, PLAG1 rearrangements, and loss of SMARCB1/INI-1 expression^[^14,15^]^. However, the relevance of these alterations in intraosseous lesions remains uncertain due to the extremely small number of reported cases. Nonetheless, molecular analysis may offer additional diagnostic value and could inform future therapeutic strategies.

Intraosseous MEC is extremely rare, with only isolated case reports and small series described to date^[^1,10^]^. Most reported tumors arise in long bones such as the femur, tibia, and humerus, and typically present with nonspecific pain or progressive swelling, frequently mimicking benign or intermediate-grade bone lesions. These nonspecific clinical and radiologic features often contribute to delayed biopsy, as noted in prior reviews[1]. Although histologic features resemble those of soft-tissue MEC, available reports suggest a relatively aggressive clinical course in bone, with high rates of local recurrence and pulmonary metastasis despite wide resection^[^1,3^].^ Epidemiologic analyses also indicate poor overall survival in MEC more broadly, with 5-year survival rates estimated at approximately 40%–60%^[^11,16^]^.

Surgical resection with negative margins remains the mainstay of treatment for MEC. As demonstrated in studies by Wang *et al* and Chamberlain *et al*, complete surgical excision is associated with improved outcomes in both soft tissue and head and neck MECs[16]. In the patient, wide resection of the proximal humerus and reconstruction with a vascularized fibular graft were performed, and surgery was deemed absolutely essential. Nakatsu *et al* similarly emphasized surgery as the primary treatment modality for respectable MEC, particularly of the parotid gland[17].

The role of radiotherapy remains controversial, especially in osseous MEC. Preoperative radiotherapy has been employed in unresectable cases or when surgery is contraindicated, sometimes resulting in a significant reduction of viable tumor cells[8]. Postoperative intensity-modulated radiation therapy (IMRT) has been used in soft tissue cases to reduce recurrence risk, although its utility in primary bone MEC is unproven[17]. In the present case, adjuvant radiotherapy was omitted because surgical margins were negative and the reconstructed fibular graft was considered susceptible to radiation-induced damage.

Chemotherapy is generally reserved for inoperable, metastatic, or high-risk cases. Regimens based on doxorubicin and ifosfamide have shown limited efficacy, though combination with proton beam therapy has demonstrated potential for prolonged survival in individual cases[18]. Chamberlain *et al* reported partial responses to systemic chemotherapy in some patients with soft tissue MEC, but overall responses remain modest[9]. In the present case, perioperative chemotherapy with paclitaxel (200 mg/m^2^) and carboplatin (AUC 5) was administered both pre- and postoperatively due to the prolonged pre-diagnostic course and concern for occult dissemination. While no recurrence or metastasis was observed for one year, the patient subsequently developed pulmonary metastases.

At the time of metastasis, the patient received immune checkpoint inhibitor therapy with nivolumab (240 mg every two weeks); however, disease progression could not be controlled. Although some anecdotal reports suggest limited efficacy, the role of immune therapy in MEC remains uncertain and warrants further investigation. While this approach was not yet established at the time, next-generation sequencing has since become more widely available, and molecular profiling is now considered a promising tool for guiding individualized systemic treatment. Although this was not feasible during the patient’s initial management, it should now be regarded as standard practice. This case underscores three important lessons: first, early biopsy is critical for atypical bone lesions; second, interdisciplinary collaboration is essential in managing rare tumors such as MEC; and third, molecular diagnostics should be integrated early to inform treatment strategies. Despite aggressive multimodal therapy, delayed diagnosis may have contributed to disease progression in the patient. Moving forward, improved awareness, earlier intervention, and comprehensive diagnostic workup are key to better outcomes in this rare but aggressive malignancy.

## Conclusion

The authors encountered a case of MEC occurring in the humerus, a highly rare location, which took 2 years to diagnose. Since most MEC cases are salivary gland carcinomas, thorough examination of the head and neck, as well as collaborative treatment planning with otolaryngology and medical oncology departments, is essential, and multidisciplinary treatment is recommended. However, we believe that early diagnosis and prompt treatment intervention are of utmost importance.

## Patient perspective

The patient’s parents reflected that, given the importance of their child’s health, a biopsy and active treatment should have been pursued from the beginning rather than 2 years of follow-up observation. They emphasized that early tissue diagnosis might have provided a better opportunity for timely intervention.

## Data Availability

No datasets were generated or analysed during the current study.

## Artificial intelligence

During the preparation of this manuscript, generative AI (ChatGPT, OpenAI, GPT-5, accessed September 2025) was used solely to improve English grammar and style.

All scientific content, clinical interpretation, and final revisions were performed and verified by the authors, who take full responsibility for the integrity and accuracy of the manuscript.
